# Primordial and primary prevention programs for cardiovascular diseases: from risk assessment through risk communication to risk reduction. A review of the literature

**DOI:** 10.6061/clinics/2016(11)09

**Published:** 2016-11

**Authors:** Inês Lancarotte, Moacyr Roberto Nobre

**Affiliations:** Hospital das Clínicas da Faculdade de Medicina da Universidade de São Paulo, Instituto do Coração, Equipe de Epidemiologia Clínica e Apoio à Pesquisa, São Paulo/SP, Brazil

**Keywords:** Health Promotion, Cardiovascular Diseases, Risk Factors, Risk Assessment, Communication, Comprehension

## Abstract

The aim of this study was to identify and reflect on the methods employed by studies focusing on intervention programs for the primordial and primary prevention of cardiovascular diseases. The PubMed, EMBASE, SciVerse Hub-Scopus, and Cochrane Library electronic databases were searched using the terms ‘effectiveness AND primary prevention AND risk factors AND cardiovascular diseases’ for systematic reviews, meta-analyses, randomized clinical trials, and controlled clinical trials in the English language. A descriptive analysis of the employed strategies, theories, frameworks, applied activities, and measurement of the variables was conducted. Nineteen primary studies were analyzed. Heterogeneity was observed in the outcome evaluations, not only in the selected domains but also in the indicators used to measure the variables. There was also a predominance of repeated cross-sectional survey design, differences in community settings, and variability related to the randomization unit when randomization was implemented as part of the sample selection criteria; furthermore, particularities related to measures, limitations, and confounding factors were observed. The employed strategies, including their advantages and limitations, and the employed theories and frameworks are discussed, and risk communication, as the key element of the interventions, is emphasized. A methodological process of selecting and presenting the information to be communicated is recommended, and a systematic theoretical perspective to guide the communication of information is advised. The risk assessment concept, its essential elements, and the relevant role of risk perception are highlighted. It is fundamental for communication that statements targeting other people’s understanding be prepared using systematic data.

## INTRODUCTION

The aging of the population, which has occurred as a result of the increased population and life expectancy, has led to an increase in the mortality due to noncommunicable diseases (NCDs). According to estimates from the World Health Organization in 2012, 68% of the 56 million global deaths were caused by NCDs, of which 46% were due to cardiovascular diseases (CVDs) 1. In Brazil, as stated by the Ministry of Health, NCDs were the cause of a large proportion of deaths from 2009-2013; CVDs were the main cause of death, at nearly 30%, of which 20% were due to ischemic heart disease, cerebrovascular disease, and high blood pressure 2.

NCDs have a long natural history, a long latent period, and a slow, lengthened and continuous clinical course, among other features, and result from exposure to a variety of risk factors 3. According to the World Health Organization, nearly a third of the world’s deaths can be attributed to ten risk factors, and the most frequent ones, e.g., high blood pressure, tobacco use, high blood glucose, high blood cholesterol, physical inactivity, overweight, and obesity, are related to the development of NCDs 4. Current knowledge has thus established the value of primordial prevention actions, which aim to prevent the development of disease risk factors, and of primary prevention actions, which aim to modify existing risk factors to prevent the development of diseases. These preventive approaches have become the basis of intervention community programs that focus on promoting cardiovascular health and preventing CVDs; however, scientific evidence is needed to demonstrate the effectiveness of the community approach to promoting public health 5,6.

Systematic reviews on the effectiveness of multiple risk factor interventions for preventing CVDs suggest that they may be effective for people at high risk of CVDs, may not be particularly effective for people at low risk of CVDs, and may achieve favorable changes in overall CVD risk, among other findings; however, considerable uncertainty remains 7,8. Methodologic differences in the design or analysis may account for the lack of successful outcomes. A better understanding of which interventions are effective and further research on the most effective and efficient ways to change the health behavior of populations are needed to improve the outcomes of future interventions 5.

Accordingly, the aim of this study was to identify and reflect on the methods employed in studies focused on intervention programs targeting the primordial and primary prevention of CVDs.

## METHODS

### Search strategy and study selection

**Figure 1:**
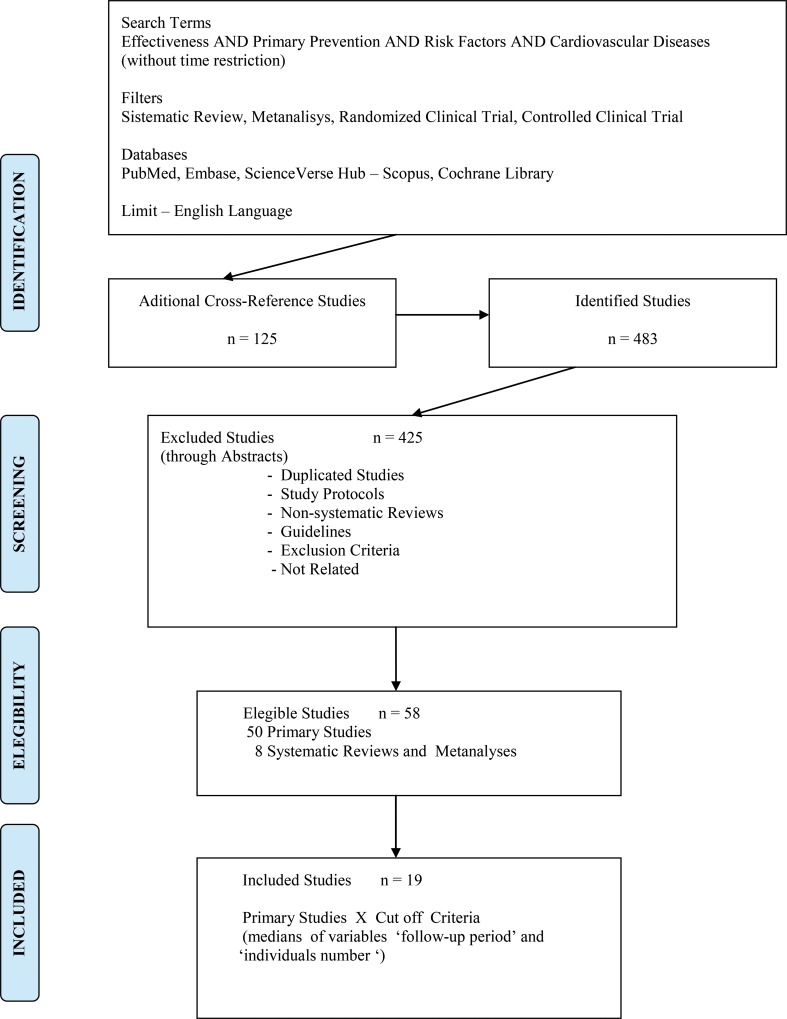
Flow chart for study selection.

The PubMed, EMBASE, SciVerse Hub-Scopus, and Cochrane Library electronic databases were searched using the terms ‘effectiveness AND primary prevention AND risk factors AND cardiovascular diseases’ for systematic reviews, meta-analyses, randomized clinical trials and controlled clinical trials limited to studies in the English language. After identification and screening, 50 primary studies and eight systematic reviews and meta-analyses met the eligibility criteria. The medians of the variables ‘follow-up period’ and ‘individual number’ in the primary studies were 24 months and 1174 individuals, respectively, and were considered criteria for study inclusion (Figure 1).

### Eligibility criteria

Population – adults exposed to cardiovascular risk factors, with or without a diagnosis of cardiovascular disease;

Intervention – community- or individual-level educational program, with or without therapeutic features, focused on cardiovascular risk factors: smoking, dietary behaviors, regular physical activity, and risk perception associated with overweight, arterial hypertension, metabolic disease, and familial inheritance;

Control – adults exposed to cardiovascular risk factors, with or without a diagnosis of cardiovascular disease, who were not exposed to intervention;

Outcomes – change in attitudes, knowledge, behavior, perceptions, and biologic measures.

### Exclusion criteria

Educational interventions targeting specific groups of diseases, conducted at work sites or second and tertiary healthcare units, or based only on electronic media.

Descriptive analyses and study syntheses of strategies, models, frameworks, applied activities, and measurement of variables were conducted, referring to studies of public health researchers affiliated with the University of Oxford.

As this article was based on studies available in the public domain, application to an Ethical Committee was not required.

## RESULTS

A community approach was employed by seven studies 9,10,11,12,13,14,15, applying principles from the social learning theory, community activation, the stage theory of innovation, the theory of planned behavior, the PRECEDE-PROCEED model, and other methods aiming to change both individual behavior and the environment, organizations, and policies to support individuals’ heathy choices. The activities included educational programs on factors related to cardiovascular risk topics provided through multiple educational channels and instruments, community organization and activation, creation of social and institutional support for educational goals, and environmental changes; some studies conducted risk factor screening and targeted improvements in preventive services.

Ten studies chose a community and individual strategy 16,17,18,19,20,21,22,23,24,25 based on social learning, diffusion of innovation, social development, persuasive communication, and models involving community leaders and institutions. These studies shared similar hypotheses, although with distinct characteristics, about the roles of individuals, communities, and the physical and social environment. The activities included integration of occupational health and primary care services, an individual approach to health care services, promotion of healthy lifestyle campaigns, continuous training programs for health teams, and establishment of guidelines for diagnostic procedures, treatment, and healthy lifestyle counseling for application in health services, as well as environmental interventions.

The theoretical foundations of the two studies applying an individual strategy 26,27 were the stages of change model, motivational interviewing, and behavioral therapy, and the activities implemented were comprehensive individual care and healthy lifestyle counseling in general practice centers (Table 1).

Heterogeneity was observed between the studies regarding the methodology of the outcome evaluation, not only in the selected domains but also in the indicators used to measure the variables (Table 2).

Other features of the studies were noted: a predominance of repeated cross-sectional survey design, the use of rural and urban settings, the inclusion of communities with high rates of poverty and low educational levels, diverse age groups and variability related to the randomization unit when randomized procedures were implemented as part of the sample selection criteria. Furthermore, specifics related to variable measures, limitations, and confounding factors were observed (Tables 3 and 4).

## DISCUSSION

The observed heterogeneity between studies is a fundamental issue. Researchers examined the results of programs targeting primordial and primary prevention of CVD and attempted to identify the determinants of their success or failure. These determinants included specific population characteristics, matching of intervention and control communities, and the characteristics, exposure time, follow-up length, and evaluation method of the intervention. In addition, they addressed two questions: “what type or model of intervention is the most effective in achieving improvements in the cardiovascular health of the population” and “which is the best evaluation program method” 5,8.

### Study Design

Most studies included in this article employed a cohort study and/or a repeated cross-sectional design.

Although randomized controlled trials are considered the gold standard for assessing the effectiveness of certain types of interventions, there are several restrictions to their use when evaluating health promotion initiatives 28. To assess the impact of community trials, studies can adopt a cohort design, a repeated cross-sectional survey design, or both simultaneously. The results based solely on a cohort study may not be representative of the target population, even if the population of interest comprises individuals residing in the community during the intervention, because the cohort sample is typically a self-selected subset of a group that is willing to be followed. Survey designs should preferably comprise independent samples with the same age and socioeconomic distribution that are ideally randomized and representative of the community 29,5.

### Strategies

Two main strategies were employed by the studies included in this review, each with its advantages and disadvantages.

Individually targeted interventions may be of limited use in community programs because the participation of individuals is generally low, leading to a small impact at the population level. In community interventions, well-designed mass media campaigns are usually effective for increasing basic knowledge but ineffective for correcting misconceptions, and public policy changes probably achieve the best impact concerning cost effectiveness. There was no agreement regarding the duration of exposure to the intervention activities, as the amount of time depended on the nature of the intervention and the characteristics of the target population. One important issue when conducting community intervention programs is the intervention’s sustainability, which is directly related to several factors. In brief, the simpler and cheaper an intervention is, and the higher the proportion of the community population covered by the intervention, the more sustainable a program will be. Additionally, sustainability is indirectly related to the intervention intensity; although the intensity should be high enough to produce changes 29, increased intensity usually leads to higher costs, more difficulty and often burnout among workers and participants.

### Theories and Frameworks

The studies in this review applied different theories and frameworks that aimed to explain individual behavior and trends within populations.

The social learning^1^ theory states that behavior change can be achieved through intense exposure to ideal or archetype models. Furthermore, it considers the influence of personal experience, observed or otherwise transferred, and the important role of self and group efficacy in changing behaviors in addition to the necessity of a supportive social setting and the development of skills to maintain new attitudes and practices.

The theory of planned behavior^2^ assumes that individuals are typically rational and systematically use the information available to them. In this theory, individuals progress through several steps to achieve behavior change – from awareness, attitudes and knowledge acquisition to motivation, skill development and action. Additionally, this theory states that the prevailing subjective norms in the community have a substantial impact on the health-related behaviors of the individuals and that self-management skills have to be learned to maintain adapted behaviors.

Persuasive communication^3^ aims to convince individuals to be more responsible for their own health through a seven-step procedure: reviewing reality, analyzing values, surveying the sociocultural situation, mapping a mental matrix, focusing on target themes, constructing communication, and evaluating the effectiveness.

The PRECEDE-PROCEED^4^ model of educational interventions is a framework that encompasses several dimensions of health and includes a large number of health professionals in planning and managing health educational programs. The initial phases, namely social, epidemiological, behavioral/environmental, educational/organizational, and administration/policy assessment, are followed by program implementation and an evaluation of its process, impact, and outcome.

Social market^5^ theories are based on the marketing orientation concept, which states that the main task of an organization is to determine the needs and demands of a population and to address these needs through design, communication, pricing, and the delivery of appropriate and competitively viable products and services. To organize preventive health services, the audience has to be defined, messages have to be developed, and the most effective channels for acceptance have to be selected. These theories combine and apply elements of the theories and frameworks described above.

The stages of change^6^ model emphasizes the importance of cognitive processes and the concept of self-efficacy and assumes that individuals progress through the following stages during the change process: precontemplation, contemplation, preparation, action, maintenance, and occasionally relapse.

The diffusion of innovation^7^ theory provides an understanding of how new ideas or behaviors are introduced and accepted by a community. This theory states that individuals advance through different stages-awareness, interest, persuasion, decision, and adoption-before changing their behavior; thus, individuals adopt new behaviors at different rates and respond to different methods of intervention. This model is similar to that of stages of change concerning the assumption that individuals progress through several stages before achieving behavior change. The main distinction between the two is that the stages of change model focuses on behavior change at the individual level, whereas diffusion of innovation focuses on behavior change in a population 5,30.

### Interventions

Although several approaches can be employed to develop intervention activities, as shown by the studies included in this review, the essential aim of these activities is to communicate risk. This communication includes a challenging process of ensuring that risk assessments and risk management information are understandable to individuals, community groups, and professionals engaged in intervention activities.

To make reasonable decisions, individuals have to understand the risks and benefits associated with alternative courses of action, the limits of their own knowledge and the various recommendations of experts. Health risk decisions are influenced not only by cognitive processes and objectively communicated information but also by emotions, individual differences, culture, and social processes; however, it is important to ensure a correct understanding to prompt and encourage people’s ability to think about their decisions.

Individuals who provide health information should have an understanding of what the targeted population knows, what they need to know, and how they should interpret the messages; a systematic theoretical perspective should guide how information is communicated. Studies point to three main approaches to communicating information:

One approach is to use a mental model analysis,^8^ which addresses differences between lay mental models and expert mental models. This analysis enables the identification of lay beliefs that would not have occurred to the experts, reduces the chances of omitting critical concepts and minimizes the clutter created by irrelevant information; furthermore, it illustrates the terms that laypeople use to express their beliefs.

Additionally, calibration analysis can be conducted to provide people the appropriate degree of confidence in their beliefs regarding situations in which they maintain false beliefs, as these could lead to incorrect behavior, or in which they lack confidence in the right beliefs. Confidence in the appropriate beliefs is needed to perform desired actions. Misconceptions can lead to incorrect conclusions even among presumably well-informed people, therefore deserving special attention in communication.

Finally, value-of-information analysis^9^ provides insight into the type of information that would have the largest possible impact on future decisions.

Once the message has been selected, it must be presented in a comprehensible way. Accordingly, terms that people use to understand concepts and mental models that people use to combine concepts must be included; In addition, results of research on text comprehension must be considered.

Effective risk communication can help people reduce their health-related risks and can help them obtain more benefits in response to risks to which they are exposed. Ineffective communication may not only fail to provide these advantages but may also lead to inappropriate decisions by omitting key information or not arguing against false beliefs, even leading to opportunity costs. Poor communication can create a larger impact on public health than the risks they aim to describe by causing undue alarm or complacency.

As all individuals have experience in dealing with risks, eliciting others’ beliefs may seem straightforward. However, this is often not the case; communicators’ intuitions about people’s risk perception cannot be trusted – there is no alternative to empirical validation 31.

### Risk assessment and risk measurement

In addition to the observed heterogeneity between studies regarding the measurement of outcomes, there were differences in risk perception.

A risk assessment is the systematic scientific characterization of potential adverse effects caused by human exposures to dangerous agents or situations; both quantitative and qualitative estimates, as well as strength of the evidence, are essential to risk characterization. A fundamental issue in risk assessment is risk perception, which is an individual’s degree of understanding of health-related risks. Individuals react very differently to information about harmful situations – one event can be accepted by one individual and not accepted by another, and understanding these responses is essential to developing risk management options 32.

### Quantitative estimates

Studies show that lay estimates of risk are subject to biases. Few studies directly associate these biases with inappropriate risk decisions or suggest that people wait for accurate information to establish their decision models. Accurate risk estimates are necessary but not sufficient for effective decisions, as estimates alone do not inform people about what actions are possible, what objectives are worth pursuing, nor what risks are worth concern. Other characteristics of the quantitative perception of risks have been noted by experts: the internal consistency of relative frequency estimates, influence of anchors provided by investigators, less dispersion of subjects’ estimates in statistical evaluations, miscalibration of confidence judgements, and availability bias. Availability bias implies that the more visible an event is, through personal or reported media experiences, the higher an estimate of risk it will receive, and this relationship seems to reflect a general tendency to estimate the frequency of events by the ease with which they are remembered or imagined. Other studies point to response mode problems: researchers’ reliance on verbal quantifiers to communicate and elicit risk estimates – e.g., terms such as “rare” or “very likely” mean different things to different people and even to the same people in different contexts; nonlinear relationships between quantitative and qualitative scales; the rather insensitive effect of the provided anchors, which are employed with the assumption of improving peoples’ performance regarding the correct range of estimates; and the dangers of applying a single response mode 31.

Assessing the accuracy of lay risk estimates relies not only on a proper response model but also on a pattern against which responses can be compared. Peoples’ performance might vary widely and be more difficult to evaluate when they are faced with risks that have an unclear magnitude; additionally, for many decisions, peoples’ understanding of population-wide risks is less relevant than their understanding of personal risks. Investigators have identified a perceived invulnerability, i.e., an optimism bias, when they evaluate how individuals judge their risks in relation to others in the same circumstances and in situations under some personal control – most people perceive that they face less risk than others 31.

Studies providing measures of risk perceptions assume that people define risk as the probability of an adverse event occurrence. Nevertheless, observations of scientific practice show that risk can be presented through a variety of meanings, even among professionals: the characterization of risk through a discrete instead of a continuous descriptor or alternatively through a safe or unsafe descriptor conveys rather little information, and without more detail, one does not know what the investigator means by a certain descriptor. Studies point to different risk definitions as a partial cause of the inconsistency between investigators and laypeople regarding the magnitude of risks in society.

The multidimensional nature of risk affects how it is judged, which means that although hazards may be similar in many ways, they may evoke quite different responses. Much research and speculation has been applied to test hypotheses that lead to a descriptive theory of risk perceptions, a prescriptive guide to risk decisions or a scheme for predicting the public’s response to new hazards or hazard reductions.

Regarding the role of perception about risk perceptions in public health, one assumption is considered fundamental: statements targeting other people’s understanding must be constructed using systematic data because people can be harmed by inaccuracies in their risk perceptions and by inaccuracies in other people’s beliefs about risk perception, particularly those of groups who communicate risks, such as health professionals 31.

### Qualitative assessment

Scientific estimates of the magnitude of risk rely on a comprehensive specification of the conditions under which it is observed; any other scenario will generate answers related to the perception each individual has about the value of each missing detail. Ambiguous events enable responses to different questions and thus result in ambiguous responses. When people are asked to answer questions they do not understand, any relationship between their related beliefs and respective behavior will tend to be blurred and lead observers to false conclusions regarding how the information-transmission activity was developed.

In addition to the methodological importance, the details people infer can be particularly interesting and illustrate intuitive theories about risk that people invoke in personally meaningful ways when facing tasks 31.

## IMPLICATIONS FOR PRACTICE

Current evidence supports the notion that complementary strategies must be employed to improve the cardiovascular health of the population 33:

- Individual approaches, which target healthy lifestyles and drug treatment when necessary, should be implemented while considering the following: that medical knowledge is based on biomedical rationality and is thus limited in addressing the complexity of the health-sickness process and that to be comprehensive, interventions focused on health promotion and on disease control should incorporate the autonomy, values, and preferences of the subjects regarding technical knowledge 34;

- Healthcare system approaches that encourage, facilitate, and reward healthcare providers’ efforts to improve health behaviors and health factors can also be employed; and

- Population approaches that target changes in lifestyle can be conducted in schools, worksites, and communities and can include the development of public policies to support lifestyle changes.

## IMPLICATIONS FOR RESEARCH

Systematic qualitative studies that focus on information and communication and evaluate how participants perceive and respond to interventions could be of great value in shaping future interventions.

^1^ Bandura A. Social learning theory. Englewoods Cliff, NJ: Prentice-Hall; 1977.

^2^ Ajzen I and Madden TJ. Prediction of goal directed behaviour, attitudes, intentions and perceived behavioural control. Journal of Experimental Social Psychology. 1986;22:414-53.

^3^ McGuire WJ. Public communication as a strategy for inducing health promotion behavioural change. Preventive Medicine. 1984;13:299-319.

^4^ Green L and Kreuter M. Health promotion planning: an educational and environmental approach (2nd edn). Palo Alto, CA: Mayfield Publishing; 1991.

^5^ Kottler P and Clarke RN. Marketing for health care organizations. Englewood Cliffs, NJ: Prentice-Hall; 1987.

^6^ Prochaska JO, Di Clemente CC, Norcross JC. In search of how people change: applications to addictive behaviours. Am Psychol 1992;42:1102-14.

^7^ Rogers EM. Diffusion of Innovations, 3rd edition. New York: Free Press; 1983.

^8^ Term applied to intuitive theories related to predictions in various circumstances

^9^ Term applied to techniques that ascertain the sensitivity of decisions to different information

## AUTHOR CONTRIBUTIONS

Lancarotte I was responsible for the literature review, data collection, synthesis and analysis of results and manuscript writing. Nobre MR was responsible for the conception and methodologic control of the study.

## Figures and Tables

**Table 1:** Characteristics of the studies by strategies, theories, models and activities.

Author/Year	S	Theory and Model	Activities
Brownson RC et al., 1996	C	Planned approach to community health model, social learning theory and stage theory of innovation. Coalition development through involvement of local leaders, community groups and local health agencies.	Walking clubs, aerobic exercise classes, heart-healthy cooking demonstrations, community blood pressure and cholesterol screenings and cardiovascular education programs.
Tudor-Smith C et al., 1998	C	Health promotion methods directed toward both changing health behaviors in individuals and achieving environmental, organizational and policy changes that support healthy choices. The study drew on the experiences of other community-based risk reduction programs for cardiovascular disease.	Television series about healthy heart-related themes, food labeling and nutrition education with a major grocery retailer, a restaurant and canteen scheme to increase the availability of healthy food choices and smoke-free areas, and a worksite health promotion program.
Puska P et al., 1983	C	Behavioral-social model of community intervention: improved preventive services to help people identify their risk factors and to provide appropriate attention and services; information to educate people about the relationship between behaviors and their health; persuasion to motivate people and to promote the intention to adopt the healthy action; training to increase the skills of self-management, environmental control and necessary action; social support to help people maintain the initial action; environmental change to create opportunities for healthy actions and improve unfavorable conditions; community organization to mobilize broad-range changes in the community to support the adoption of new lifestyles in the community.	Educational activities through the mass media – production of educational material about health and support to campaigns and community meetings; training programs to local staff – doctors, nurses, social workers, teachers, volunteer organization representatives and informal leaders; reorganization of preventive services through formal decisions, training, demonstrations, materials and guideline provision; activities with community organizations – medical and women’s associations, sport clubs, food industries and groceries; monitoring project development – information systems to assess the intervention.
Nafziger NA et al., 2001	C	Community organization with leaders from businesses, churches, educational facilities, government offices and others; implementation of the health promotion programs according to each community’s needs.	Risk factor screenings, physical activity events, programs in schools, restaurants and groceries, and the development of mass media communication strategies.
Carleton RA et al., 1995	C	Social learning theory. The focus was on helping individuals adopt new behaviors and on creating a supportive physical and behavioral environment.	Three dimensions of activities: risk factors – elevated blood cholesterol and blood pressure, cigarette smoking, obesity and physical inactivity; behavior change – promoting awareness and agenda setting, providing training in behavior skills, developing social support and strategies for maintenance of new behaviors; community activation – focus on individuals and their surrounding groups and organizations in addition to programs available to all community members.
Huot I et al., 2004	C	Social learning, planned behavior approach to communities, social marketing, persuasive communication, and diffusion of innovation theories, community development strategies and the PRECEDE-PROCEED model. Program development in elementary schools, with the assumption that adults would be reached through children’s activities; public health approach, community-based and multifactorial programs, and involvement of broad segments of the population and local organizations.	Classes targeting nutrition, physical activity and smoking prevention; invitation to parents to participate in school-based and community activities; articles in local newspapers; conferences, cooking classes, healthy food tasting, distribution of health recipe booklets, games and tips in local stores and restaurants for healthy food choices; walking clubs; screening sessions for hypertension and hypercholesterolemia.
Winkleby MA et al., 1996	C	Learning theories combined with community change theories to reach individuals and collaborate through changes with community institutions.The project was designed to create a self-sustaining health promotion structure based on community organizations that remained at the end of the intervention.	Intervention targeted all residents through multiple educational channels: interpersonal meetings, classes and correspondence courses, distribution of print media products through direct mail and worksites and medical care providers, programs in mass media, and materials targeting low-literacy and low-income individuals.
Nguyen QN et al., 2012	C + I	Development of the program through two components – one targeting local hypertensive patients and the other targeting the local general population through three interactive approaches: comprehensive information education and communication, standard protocols at the community health station and a continuous training program to improve the capacity of the local cardiac care team.	For hypertensive individuals: monthly check-ups, drug therapy and individual lifestyle modification advice. For healthy adults in the entire community: periodic lifestyle promotion campaigns via broadcasting, leaflets or meetings with messages focused on smoking cessation, reduction of alcohol consumption, increase in physical activity and healthier diets (encouraging reduction in salt and consumption of vegetables and fruits).
Schuit AJ et al., 2006	C+ I	The model postulates that a reduction in cardiovascular diseases can be achieved through changes in related risk behaviors and that behavioral changes are expected to result from changes in individuals’ psychosocial determinants – awareness, attitudes, social influences, self-efficacy expectations and stages of change – through sufficient, tailored and effective activities with community participation, intersectorial collaboration, adjustment to the current situation, long-term continuation of the project, a multi-media and multi-method strategy and environmental changes.	Personal and group sessions with written, tailored information communicated via mass media; computer-tailored nutrition education, nutrition education tours in super-markets, food labeling, promotion of physical activity, regional campaign to promote physical activity among individuals over 55 years, television programs, walking and bicycling clubs, walking and cycling campaigns, stop-smoking campaigns; activity development according to the characteristics of the target groups.
Wendel-Vos GCW et al., 2009	C + I	The model postulates that a reduction in cardiovascular diseases can be achieved through changes in related risk behaviors and that behavioral changes are expected to result from changes in individuals’ psychosocial determinants – awareness, attitudes, social influences, self-efficacy expectations and stages of change – through sufficient, tailored and effective activities with community participation, intersectorial collaboration, adjustment to the current situation, long-term continuation of the project, a multi-media and multi-method strategy and environmental changes.	Personal and group sessions with written, tailored information communicated via mass media; computer-tailored nutrition education, nutrition education tours in super-markets, food labeling, promotion of physical activity, regional campaign to promote physical activity among individuals over 55 years, television programs, walking and bicycling clubs, walking and cycling campaigns, stop-smoking campaigns; activity development according to the characteristics of the target groups.
Kottke TE et al., 2006	C + I	Social modeling and diffusion of innovation theories; the North Karelia Project was the primary model of practical application. Study hypothesis – supposition that sustained behavior change requires both the stimulation of individuals to attempt behavior change and a change in the physical and social environment to support individuals who are trying to change.	Television programs, radio interviews, newspaper feature articles in the model of ‘behavioral journalism’-intervention techniques that publicize the healthy behavior of real community people. Competitions – smoking cessation, physical activity and weight control. Environmental improvement – creating smoke-free restaurants, implementing a menu-labeling program for restaurants, cafeterias and other suppliers of ready-to-eat food and advocating for the construction of multi-use trails as a way to increase public opportunities for daily physical activity.
Lupton BS et al., 2002	C + I	Learning by doing rather than traditional health promotion; local empowerment, which emphasizes the potential of the individual and the community to take responsibility in making decisions, prioritizing and achieving power over one’s own destiny.	Safety-at-work programs and occupational health services were established in cooperation with trade unions and integrated into the public primary care services. First phase – improving work conditions; second phase – individual counseling about diet, smoking and physical activity as part of ordinary consultations with general practitioners, public health nurses and occupational health services.
Lupton BS et al., 2003	C+ I	Community empowerment – to influence the whole population to be more health conscious, to mobilize the inhabitants to participate in health-promoting activities and to change the environmental factors influencing health.	Aerobic classes for ladies, physical training for individuals with heart disease, walking, volleyball and football competitions, dancing meetings, and swimming lessons. Healthy recipes, menus based on local food tradition and cooking classes. Smoke-free rooms in public buildings. Distribution of manual with suggestions for health-promoting improvements to schools, voluntary organizations and local public administration; local newspapers, radio and television were used throughout the intervention period. Establishment of guidelines for local general practice regarding individual counseling on quitting smoking, following heart-favorable diets and engaging in physical activity.
Record NB et al., 2000	C + I	Approach using screening, counseling, referral, follow-up, continuity, physician involvement, and community activism in addition to educational activities targeted to individuals, particularly those with low literacy, the community and health professionals.	Nurse-mediated community program – personal and family history, symptoms, medications and lifestyle; measurements of weight, blood pressure and cholesterol and personal counseling.
Hoffmeister H et al., 1996	C + I	Social learning and diffusion of innovation theories. Methods based on experiences of other community studies. Prevention programs focused on improving health knowledge, awareness, attitude and behavior.	Health nutrition: campaigns at community events, restaurants, supermarkets and schools, ‘weight reduction’ courses, seminars on nutritional topics and preparation of healthy foods, availability of low-salt, low-fat and low-calorie products, and encouragement for higher consumption of vegetables and cereal products. Physical activity: recreational sports events. Smoking habits: anti-smoking campaigns and establishment of non-smoking areas in public places.
Luepker RV et al., 1994	C + I	Social learning and persuasive communication theories and models for involvement of community leaders and institutions.	Campaigns via the mass media, training programs for primary care physicians and other health professionals. Screening, education and counseling for adults and direct education programs for children about health-enhancing behaviors. Community involvement in environmental change programs.
Weinehall L et al., 2001	C + I	Primary prevention in the community as a social change process.	Annual comprehensive health examinations with counseling by family physicians, nurses and dieticians. Messages about lifestyle factors in local associations, sports clubs, media and food retailers; health education activities through theater, music and informal meetings.
Wood DA et al., 2008	I	Stages of change model and various methods to increase motivation, overcome barriers and develop strategies. Commitment to increase the population’s quality of life through reducing the impact of cardiovascular disease. Program objective – to help individuals at high risk of developing cardiovascular disease achieve lifestyle, risk factor and therapeutic goals defined in the ‘Joint European Societies’ guidelines.	Nurse assessment: family lifestyle, risk factors, drug treatment, health beliefs, anxiety, depression, illness perception and mood. Personal record card for lifestyle and risk factor targets. Counseling for adopting a healthy lifestyle with family and health professional support. Management of blood pressure, lipids and blood glucose.
Mortality rates. MRFIT, 1990	I	Behavioral therapy: functional analytical approach to clinical data and treatment of observed activities	Clinical evaluation: medical history and examination, laboratory tests, electrocardiograms and submaximal graded treadmill exercise. Encouragement to change eating habits – reductions in intake of saturated fats, total fats and cholesterol and moderate increases of polyunsaturated fats, weight reduction and cessation of tobacco use.

S – Strategy, C – Community level, I – Individual level.

**Table 2:** Characteristics of studies by domain and measurement approach of the selected variables.

Author/Year	Variables – Domain and measurement approach
Brownson RC et al., 1996	Attitude and Behavior Leisure-time physical activity, Current smoker, Consumes 5+ servings of fruits and vegetables per day, Cholesterol checked in past 2 years, Overweight
Tudor-Smith C et al., 1998	Attitude and Behavior Health-enhancing behaviors: Consumes chicken/other poultry ≥2 days/week, fish ≥2 days/week, fresh fruit ≥4 days/week, green vegetables/salad ≥4 days/week, Mainly uses skimmed/semi-skimmed milk at home, Mainly uses whole meal bread, Smokers who agree that their present level of smoking is harmful to their health, Smokers who have made a serious attempt to quit smoking in the 12 months before the survey, Daily smokers visiting their general practitioner in the 12 months before the survey who were advised to cut down or stop smoking, Engages in moderate or strenuous activity ≥2 times/week for ≥20 minutes each time Health-compromising behaviors: Mainly uses butter on bread, Consumes fried food cooked in lard/other solid fat ≥2 days/week at home, Smokes daily, Mean number of cigarettes/day smoked by daily smokers, BMI (kg/m^2^) ≥24 for women and ≥25 for men
Puska P et al., 1983	Attitude, Behavior and Biological Measures Reported daily smoking, Weight, Height, Systolic and diastolic blood pressure, Cholesterol and thiocyanate serum concentration
Nafziger NA et al., 2001	Attitude, Behavior and Biological Measures Reported arterial hypertension, Diabetes, Cigarette smoker, Sedentary, Weight, Height, Systolic and diastolic blood pressure, Waist and hip circumference, Exhaled carbon monoxide concentration, Fasting serum glucose and lipid profile, Electrocardiogram
Carleton RA., 1995	Attitude, Behavior and Biological Measures Reported smoking, Systolic and diastolic blood pressure, Weight, Height, Cholesterol serum concentration, Projected cardiovascular disease within 10 years
Huot I et al., 2004	Attitude, Behavior and Biological Measures Reported arterial hypertension, Diabetes, Hypercholesterolemia, Heart disease or other, Smoking status, Physical activity, Dietary behaviors, Self-reported weight and height Dietary behaviors – food frequency questionnaire adapted from the validated Ammerman Dietary Risk Assessment questionnaire: Global Dietary Index and Consumption Indices (for specific food groups – dairy products, meat products and major sources of fat)
Winkleby MA et al., 1996	Knowledge and Biological Measures Knowledge of cardiovascular diseases, Weight, Height, Systolic and diastolic blood pressure, Cholesterol and thiocyanate serum concentration, Exhaled carbon monoxide concentration, Coronary heart disease risk (morbidity and mortality in 12 years – Framingham)
Nguyen QN et al., 2012	Awareness, Attitude, Behavior and Biological Measures Reported current daily smoking, Heavy alcohol consumption, Physical inactivity, Salty diet, Weight, Height, Waist and hip circumference, Waist-hip ratio, Systolic and diastolic blood pressure, BMI, Prevalence of hypertension, Prevalence of obesity; Among hypertensives – Awareness, Being treated, Being monitored
Schuit AJ et al., 2006	Attitude, Behavior and Biological Measures History of coronary heart disease and smoking, Weight, Height, Waist circumference, Systolic and diastolic blood pressure, Serum cholesterol and glucose, Medication for hypertension, Medication for hyperlipidemia, BMI
Wendel-Vos CW et al., 2009	Attitude, Behavior Energy intake, Intake of saturated, polyunsaturated, and mono-unsaturated fat, Leisure-time physical activity, Walking, Bicycling, Sports, Smoking
Kottke TE et al., 2006	Awareness, Attitude, Behavior and Biological Measures Reported behavior change as a result of the program; Among smokers – Reported taking some action to quit smoking, Reported taking some action to try to lower cholesterol; If doing anything to lower cholesterol – Reported reducing fat in diet/watching diet/eating balanced diet/eating better to lower cholesterol, Reported number of fruit/vegetables and vegetable servings per day, Reported trying to increase the amount of exercise/day, Reported average minutes of physical activity/week, Reported doing any physical activity or exercise during past month, Zero tobacco use, Zero exposure to environmental tobacco smoke, Intake of 5 servings of fruits and vegetables/day; If diagnosed with coronary heart disease – Cholesterol<200 mg/dL, Systolic blood pressure<130 mmHg and Diastolic blood pressure<85 mmHg, BMI, Physical activity every day
Lupton BS et al., 2002	Attitude, Behavior and Biological Measures Daily smoking, Boiled coffee, Filter coffee, Low-fat milk, Unsaturated cooking fats, Unsaturated spreading fats, Physically active, Systolic and diastolic blood pressure, Cholesterol, BMI, Myocardial infarction risk score
Lupton BS et al., 2003	Attitude, Behavior and Biological Measures Daily smoking, Boiled coffee, Filter coffee, Low-fat milk, Unsaturated cooking fats, Unsaturated spreading fats, Physically active, Systolic and diastolic blood pressure, Cholesterol, BMI
Record NB et al., 2000	Biological Measures Age-adjusted total and heart disease death rates
Hoffmeister H et al., 1996	Attitude, Behavior and Biological Measures Smoking, Systolic and diastolic blood pressure, Total serum cholesterol and HDL, BMI
Luepker RV et al., 1994	Attitude, Behavior and Biological Measures Smoking, Physical activity, Blood cholesterol, Systolic and diastolic blood pressure, BMI, Coronary heart disease risk (according to the method of Truett et al. and coefficients by Leaverton et al.)
Weinehall L et al., 2001	Attitude, Behavior and Biological Measures Daily smoking, Total cholesterol, Systolic and diastolic blood pressure, Estimated coronary heart disease mortality risk (according to the North Karelia Project model)
Wood DA et al., 2008	Attitude, Behavior and Biological Measures Not smoking; Food habit questionnaire (validated against a 7-day diet diary): Saturated fat<10% of total energy, Fruit and vegetables>400 mg/day, Fish>20 mg/day, Fish oil>3x/week, Alcohol<30 g/day; BMI<25 kg/m2, Waist circumference≤80 cm for women and ≤94 cm for men, Physical activity (7-day activity recall) moderate intensity 30-45 minutes 4-5 times/week, Arterial pressure<140/90 mmHg or<130/85 mmHg in people with diabetes, Total cholesterol<5.0 mmol/L, LDL<3.0 mmol/L, Blood glucose<6.1 mmol/L; In patients with diabetes – Hemoglobin A1C<7%, medications as clinically indicated
Mortality rates MRFIT, 1990	Biological Measures Coronary heart disease, Cardiovascular heart disease and All-cause mortality.

BMI – Body mass index; HDL – High-density cholesterol; LDL – Low-density cholesterol.

**Table 3:** Characteristics of studies by target population, selection process and individual number.

Author/Year	Population of Intervention Sample	Population of Control Sample	Random (R) Not Random (nR)	Number of Individuals Intervention (I) Control(C)
Brownson RC et al., 1996	United States, Missouri, rural area, high rates of poverty, low educational levels – Households with working phones / M and W>18 years	Before and after intervention data and rural area Missouri ‘Behavioral Risk Surveillance System’ data	R – House phone numbers	1006 in 1990 1510 in 1994
Tudor-Smith C et al., 1998	United Kingdom, Wales – Households/Individuals aged 18-64 years	United Kingdom, Tyne and Wear, Cleveland Durham and North Yorkshire	R – Households	18538 in 1985 13045 in 1990
Puska P et al., 1983	Finland, North Karelia – M and W aged 25-59 years in 1972;aged 30-64 years in 1977; aged 25-64 years in 1982	Finland, eastern region	R – National population register	9241 in 1977 9002 in 1977 4723 in 1982 Total 22966
Nafziger NA et al., 2001	United States, New York, Otsego and Schohaire, rural area – M and W aged 20-69 years, living in area at least 6 months/year	United States, New York, Herkimer, rural area	R – House phone numbers	Cross-sectional 626 initial 548 after 5 years Panel study 424
Carleton RA et al., 1995	United States, Pawtucket – Individuals aged 18-64 years	United States, southeastern New England city	R – Households	Each survey – between 2037 and 2955 Total – 15261
Huot I et al., 2004	Canada, Montreal, St-Louis-du-Parc, urban site; Fabreville, suburban site; Rivière-du-Loup, rural region – Schoolchildren/ Schoolchildren’s parents	Canada, non-equivalent groups, as similar as possible to the experimental sites in terms of socioeconomic status, language spoken and geographical location	nR	4863 in 1993 5260 in 1997 School numbers Urban site: I-8/C-16; Suburban site: I-1/C-2; Rural region: I-10/C-9
Winkleby MA et al., 1996.	United States, California: Salinas and Monterey – Individuals aged 12-74 years	United States, California: Modesto, San Luis Obispo and Santa Maria	R – Households	1701 in 1979-1980, initial 1750 in 1985-1986, end 1801 in 1989-1990, 3 years later
Nguyen QN et al., 2012	Vietnam, Hanoi, Ba-Vi – rural community Phu-Cuong Individuals>25 years	Vietnam, Hanoi, Ba-Vi – rural community Phu-Phuong	R – Local inhabitants	I-1176; C-1131 in 2006 I-1192; C-1189 in 2009 Study unit – community
Schuit AJ et al., 2006	The Netherlands, Maastricht – M and W aged 31-70 years	The Netherlands, Doetinchem	R – Community	I: M-1187/W-1169/C: M-349/W-409 Sample size C<I
Wanda Wendel-Vos GC et al., 2009	The Netherlands, Maastricht – M and W aged 31-70 years	The Netherlands, Doetinchem	R – Community	I: M-1187/W-1169/C: M-349/W-409 Sample size C<I
Kottke TE et al., 2006	United States, Minnesota, Olmsted County – Adult residents.	Before and after intervention data and Minnesota and national ‘Behavioral Risk Surveillance System’ data	R – House phone numbers	1232 in 1999 1224 in 2000 1210 in 2001 1229 in 2003
Lupton BS et al., 2003	Norway, county of Finmark, Batsfjord – fishing village with 2500 inhabitants/all residents aged 40-62 years	Norway, county of Finmark, Loppa, Gamvik and Masoy – altogether 5000 inhabitants	nR – Community R – 15% of residents aged 20-39 years	Total 2435 in 1987 Total 1324 in 1993: I–364/C–960
Lupton BS et al., 2002	Norway, county of Finmark, North Cape – fishing village with 4000 inhabitants/all residents aged 40-62 years	Norway, county of Finmark, Loppa, Gamvik and Masoy	nR – Community R – 15% of residents aged 20-39 years	Total all in 1987 1685 in 1993/I–725/C–960
Record NB et al., 2000	United States, Maine, Franklin County –predominantly rural communities/adults.	United States, Maine, Oxford and Somerset – predominantly rural communities	nR – Community	13231 death certificates
Hoffmeister H et al., 1996	Germany, Berlin, Bremen and Stuttgart, Karlsruhe (Bruchusal and Mosbach), Traunstein (rural district) – Adults aged 25-69 years	West Germany – representative sample of the population	nR – Communities R – Individuals	I-1900/area: total 13300 – initial 1400/area: total 9800 – middle 1400/area: total 9800 – end Total 28927 C: 5000 each phase Total 15444
Luepker RV et al., 1994	United States, Minnesota, Mankato, Fargo-Moorhead and Bloomington – Adults aged 25-74 years	United States, Minnesota, Winona, Sioux Falls and Roseville (small, medium and urban communities)	nR – Communities R – Households	Cross-sectional: sample 300-500 Total 18062 Cohort – 7097 Study unit – individual
Weinehall L et al., 2001	Sweden, Västerbotten County – Norsjö community, rural/all people aged 30, 40, 50, 60 years	Sweden, Norbotten and Västerbotten – Northern Sweden MONICA Study	nR – Community R – Individuals/control area	Cross-sectional I-2288/C-4749
Wood DA et al. 2008	Denmark, Italy, Poland, Spain, the Netherlands and the United Kingdom – primary care centers/all eligible individuals aged 50-80 years and partners	Denmark, Italy, Poland, Spain, the Netherlands and the United Kingdom – primary care centers/all eligible individuals aged 50-80 years, without partners.	R – Primary care centers	I-1257/C-1128
Mortality rates. MRFIT, 1990	United States, many cities – Men aged 35-57 years with increased risk of coronary heart disease death	United States, many cities – Men aged 35-57 years with increased risk of coronary heart disease death	R – Individuals	I-6428/C-6438 Total: 12866

M – Men, W – Women.

**Table 4:** Characteristics of studies by design, follow-up, measurement, limitations and confounding factors.

Author/Year	Study design/Follow-up period	Measurement	Limitations and confounding factors (according to the authors)
Brownson RC et al., 1996	R I C-S survey, two samples/60 months	No comprehensive information on the accuracy of ‘Behavioral Risk Factor Surveillance System’ data during the study period	Potential response bias due to a lack of phone coverage of certain sociodemographic groups. Effects of national programs: National High Blood Pressure Education Program and National Cholesterol Education Program.
Tudor-Smith C et al., 1998	R I C-S survey, two samples/60 months	Brief interview (BI) Self-completed questionnaire (CI) Response rate: I – in 1985: BI=88%, CI=67% in 1990: BI=79%, CI=61% C – in 1985: BI=84%, CI=64% in 1990: BI=77%, CI=61%	Control group sample size at baseline was too small to provide sufficient statistical power for analysis. Diffusion of Heartbeat Wales projects and programs to control group was faster and to a far great extent than initially expected. Introduction of a national program. Favorable secular trends in smoking and dietary choices.
Puska P et al., 1983	R I C-S survey, three samples/120 months	In 1982, the cuff sphygmomanometer was longer than the one used in 1972/1977.	Finnish health service system and cultural factors.
Nafziger NA et al., 2001	R I C-S survey, two samples, cohort/60 months.		Cross-contamination and testing effects. Insufficient sample size.
Carleton RA et al., 1995	R I C-S survey, six samples/102 months	Response rate: 68%	National education programs, commercial marketing programs and secular trends. Time of economic difficulty – high unemployment and low incomes.
Huot I et al., 2004	R I C-S survey, two samples/48 months	Food frequency questionnaires-restricted food list, choice of frequency and difficulty remembering foods eaten in the past as well as their quantity.	Study population not representative of the adult population of the participating communities. Secular trends. Study design. Insufficient participation rates. In urban site, activities were directed mainly toward children, and parental participation was low. Cross-contamination.
Winkleby MA et al., 1996.	R I C-S survey, three samples/108 months		Positive and negative secular trends. Health promotion activities through popular press and voluntary agencies. Advent of broad-based federal programs. Antismoking legislation and education.
Nguyen QN et al., 2012	R I C-S survey, two samples/36 months	Self-reported behavioral questionnaire – recall bias. Arterial pressure measure in one visit and weight measure influenced by the harvest cycle.	Blood tests conducted in only part of the sample due to budget constraints. Epidemiological transitional status – rural populations adopting urban lifestyles. Negative effects of globalization. Hawthorne effect.
Schuit AJ et al., 2006	Cohort/60 months	Data influenced by seasonalityResponse rate: 80%	Study individuals were involved in previous monitoring studies. Age-associated changes in cohort design. Study not blinded.
Wanda Wendel-Vos GC et al., 2009	Cohort/60 months	Response rate: 80%	Study individuals were involved in previous monitoring studies. Measurements in C group were conducted over a longer time span than those of I group. Study not blinded.
Kottke TE et al., 2006	R I C-S survey, four samples/48 months	Self-reported data. Biological data from Mayo Clinic records.	Reliability of biological data differed from that of data collected in the context of high-quality research protocols. No control group. Community physicians with practices that focus on healthy lifestyles. Antismoking legislation. Aggressive marketing of low-carbohydrate/high-saturated fat diets.
Lupton BS et al., 2003	Cohort/36 months	Response rate – second invitation: I-61% and C-70%	Lack of randomization of communities, differences in lifestyle factors at baseline, secular trends, countywide intervention programs and crossover contamination. Inhabitants’ worries about high morbidity and mortality from coronary disease. Improvements in social conditions during the study period.
Lupton BS et al., 2002	Cohort/36 months	Response rate – second invitation:I and C-70%	Lack of randomization of communities, baseline differences, secular trends, countywide intervention programs and crossover contamination.
Record NB et al., 2000	Ecologic, observational, retrospective/240 months		Undetected secular trends. Concurrent initiatives.Death certificate data reliability – including possible influence of program awareness by local physicians. Program intensity measure. Economic fluctuations.
Hoffmeister H et al., 1996	R I C-S survey, three samples/84 months	Response rate: I-74.5/73.0/71.6% C-66.7/71.4/69.0%	Nationwide programs, regional preventive activities, self-help initiatives.
Luepker RV et al., 1994	R I C-S survey, seven samples Cohort/72-84 months	Response rate: Cross-sectional-78.7 Cohort-67.1%	Lack of randomization of intervention communities. Favorable secular trends in exposure to coronary heart disease risk reduction messages and activities. Cross-contamination.
Weinehall L et al., 2001	R I C-S survey, ten samples, Control – three samples/120 months	C – Participation rate: 76.7-81.3%	Intervention area with high cardiovascular disease incidence. Data collection: I – October and November C – January to April
Wood DA et al., 2008	Matched, cluster-randomized, controlled trial/41 months		Statistically underpowered – number of patients and partners was much smaller than expected, heterogeneity between patients, characteristics and pairs of centers. Knowledge of possible audit among randomized usual care centers. Characteristics of non-responders.
Mortality rates MRFIT, 1990	Cohort/120 months		Each death certificate was independently coded by two nosologists, and disagreements were adjudicated by a third nosologist.

R I C-S=Repeated Independent Cross-Sectional, I=Intervention group, C=Control group.
